# First record of the lac-producing species *Kerrianepalensis* Varshney (Hemiptera, Kerriidae) from China, with a key to Chinese species

**DOI:** 10.3897/zookeys.1061.73114

**Published:** 2021-09-28

**Authors:** Nawaz Haider Bashir, Weiwei Wang, Juan Liu, Wei Wang, Hang Chen

**Affiliations:** 1 Research Institute of Resource Insects, Chinese Academy of Forestry, Kunming, China Research Institute of Resource Insects, Chinese Academy of Forestry Kunming China; 2 The Key Laboratory of Cultivating and Utilization of Resources Insects, State Forestry Administration, Kunming, China The Key Laboratory of Cultivating and Utilization of Resources Insects, State Forestry Administration Kunming China

**Keywords:** Coccoidea, lac insects, Oriental China, taxonomy

## Abstract

Lac insects include astonishing species responsible for lac production. Lac is composed of resins, dyes, and shellac wax with significant economic importance. Previously, 11 species of the genus *Kerria* were reported from China, with the highest species diversity in Yunnan province. Another lac-producing species of the genus *Kerria*, namely *Kerrianepalensis* Varshney, is recorded for the first time in Yunnan province, China, on *Dalbergiacochinchinensis* Pierre ex Laness. (Fabaceae), a new host plant. In addition, a key to the 12 *Kerria* species recorded in China is also given.

## Introduction

Scale insects (Hemiptera, Coccoidea) are classified into 35 extant families, with more than 8300 described species to date (García Morales et al. 2016). These are phytophagous insects found in all zoogeographical realms except Antarctica (Ahmad et al. 2014). Lac insects belong to family Kerriidae, which is comprised of nine genera and 101 species worldwide (García Morales et al. 2016). Currently, the genus *Kerria* contains 29 species known in Asia (Table 1) and distributed in tropical and subtropical regions (Varshney and Sharma 2020). More than 20 species of *Kerria* have been described and recorded from India, Myanmar, Nepal, Pakistan, and Thailand. So far, 11 species of the genus *Kerria* have been reported from China (Varshney 1976; Chen et al. 2011), with *K.ruralis* (Wang, Yao, Teui & Liang) and *K.yunnanensis* (Ou & Hong) being endemic species (Chen et al. 2013).

**Table 1.** Worldwide distribution of the genus *Kerria*.

No.	Species	Distribution	Reference
1	*Kerriaalbizziae* (Green, 1911)	India, Myanmar, Sri Lanka	Varshney 1976; Chen et al. 2013
2	*Kerriabrancheata* Varshney, 1966	India	Varshney 1976
3	*Kerriacanalis* Rajgopal, 2021	India	Rajgopal et al. 2021
4	*Kerriachamberlini* Varshney, 1966	Bhutan, China, India, Myanmar, Nepal, Thailand	Varshney 1976; Chen et al. 2013
5	*Kerriachinensis* (Mahdihassan, 1923)	Bhutan, Cambodia, China, India, Myanmar, Nepal, Thailand, Tibet, Vietnam	Chen et al. 2011, 2013; Varshney and Sharma 2020
6	*Kerriacommunis* (Mahdihassan, 1923)	India	Varshney 1976
7	*Kerriadestructor* Talukder & Das, 2020	India	Talukder and Das 2020
8	*Kerriadubeyi* Ahmad & Ramamurthy, 2013	India	Ahmad et al. 2013a
9	*Kerriaebrachiata* (Chamberlin, 1923)	India, Myanmar, Nepal, Pakistan	Varshney 1976; Chen et al. 2013
10	*Kerriafici* (Green, 1903)	China, India, Pakistan, Thailand	Varshney and Sharma 2020
11	*Kerriagreeni* (Chamberlin, 1923)	China, Philippine, Thailand	Chen et al. 2013
12	*Kerriaindicola* (Kapur, 1958)	India	Varshney 1976
13	*Kerriajavana* (Chamberlin, 1925)	India, Indonesia, Malaysia	Chamberlin 1925; Chen et al. 2013
14	*Kerrialacca* (Kerr, 1782)	Azerbaijan, Bangladesh, China, Georgia, Guyana, India, Malaysia, Myanmar, Nepal, Pakistan, Sri Lanka, Thailand	Chen et al. 2013; Varshney and Sharma 2020
15	*Kerriamaduraiensis* Ahmad & Ramamurthy, 2013	India	Ahmad et al. 2013b
16	*Kerriamanipurensis* Ahmad & Ramamurthy, 2013	India	Ahmad et al. 2013b
17	*Kerriamengdingensis* Zhang, 1993	China	Zhang 1993
18	*Kerriameridionalis* (Chamberlin, 1923)	China, Philippines, Thailand	Chen et al. 2013
19	*Kerrianagoliensis* (Mahdihassan, 1923)	Bangladeshi, India, Pakistan	Varshney 1976; Chen et al. 2013
20	*Kerrianepalensis* Varshney, 1976	China, India, Myanmar, Nepal	Varshney 1976; Chen et al. 2011
21	*Kerriapennyae* Ahmad & Ramamurthy, 2013	India	Ahmad et al. 2013a
22	*Kerriapusana* (Misra, 1930)	India, Indonesia, Malaysia, Myanmar	Varshney 1976; Chen et al. 2013, 2011;
23	*Kerriarangoonensis* (Chamberlin, 1925)	China, India, Indonesia, Myanmar, Thailand	Chamberlin 1925; Varshney 1976; Chen et al. 2013
24	*Kerriaruralis* (Wang, Yao, Teui & Liang, 1982)	China	Chen et al. 2011
25	*Kerriasharda* Mishra & Sushil, 2000	India	Varshney and Sharma 2020
26	*Kerriasindica* (Mahdihassan, 1923)	Bangladesh, China, India, Pakistan	Chen et al. 2011, 2013
27	*Kerriathrissurensis* Ahmad & Ramamurthy, 2013	India	Ahmad et al. 2013b
28	*Kerriavarshneyi* Ahmad & Ramamurthy, 2013	India	Ahmad et al. 2013a
29	*Kerriayunnanensis* (Ou & Hong, 1990)	China	Chen et al. 2011

Lac insects are fully depending on their host plant and till now, more than 400 host plants have been recorded (Sharma 2017). Ber (*Ziziphusmauritiana* Lam.: Rhamnaceae), Kusum (*Schleicheraoleosa* Lour.: Sapindaceae), and Palas (*Buteamonosperma* Lam.: Fabaceae) are the common host plants for the production of lac in India (Bhatnagar et al. 2020), whereas *Acaciacatechu* Willd., *A.nilotica* Willd. ex Delile (Fabaceae), *Buteamonosperma*, *Samaneasaman* (Jacq.) Merr., (Fabaceae), and *Ziziphusmauritiana* are potential lac host plants in Bangladesh (Ferdousee et al. 2010). Lac host plants in China are *Dalbergiaszemaoensis* Prain, *D.assamica* Benth, *D.obtusifolia* Prain, *Puerariatonkinensis* Gagn. (Fabaceae), *Ficusaltissima* Blume, and *F.racemosa* L. (Moraceae) (Chen et al. 2010, 2011).

Herein, we redescribe and illustrate *K.nepalensis* Varshney, a species recorded for the first time from Yunnan province and China. We also provide a key to the 12 Chinese species of *Kerria*.

## Materials and methods

Twigs bearing *K.nepalensis* (new record) were collected by Dr Juan Liu from roadside *Dalbergiacochinchinensis* trees at Mengzi city (22°56'N, 103°32'E), Yunnan province, China, on 15 September 2020. Fresh samples of adult females were preserved in 75% ethanol. Specimens were placed in 10% KOH for few hours and rinsed in 5–8 changes of distilled water for preparation of permanent slides as described previously (Chen et al. 2008). The photographs and measurements were taken with a Keyence VHX-1000 digital microscope. Terminology mainly follows Kondo and Gullan (2007) and Ahmad et al. (2013b). All specimens are deposited in the museum of Research Institute of Resource Insects, Kunming, China (**RIRI-CAF**).

More than 10 individuals were selected for observation under electron microscope. The dehydration of specimens was accomplished by passing through a series of increasing alcohol concentrations as 30%, 50%, 70%, 80%, 90%, and 95% alcohol (Mehdizadeh et al. 2014). They were placed on a conductive resin and gilded for 60 sec in an ion plating machine (JS-1600, Beijing Htcy Technology Co., Ltd, China) and then observed under an electron microscope (TM3000, Hitachi High-Technologies Corporation, Japan). Photographs were arranged by using Adobe Photoshop 8.0.

## Taxonomy

### Class Insecta Linnaeus, 1758

**Order Hemiptera Linnaeus, 1758**

**Suborder Sternorrhyncha Amyot & Audinet-Serville, 1843**

**Superfamily Coccoidea Handlirsch, 1903**

**Family Kerriidae Lindinger, 1937**

#### Genus *Kerria* Targioni Tozzetti, 1884

##### Kerria nepalensis

Taxon classificationAnimaliaHemipteraKerriidae

Varshney, 1976

2C01C044-D889-5BEA-BEFA-C063505F6EF2

Figures 1 , 2

###### Material examined.

China: Yunnan: Mengzi city, 22°56'N, 103°32'E, 15.IX.2020, coll. Juan Liu, *Dalbergiacochinchinensis* (Fabaceae), 5 slides (10 adult ♀♀).

###### Diagnosis.

**Figure 1.**
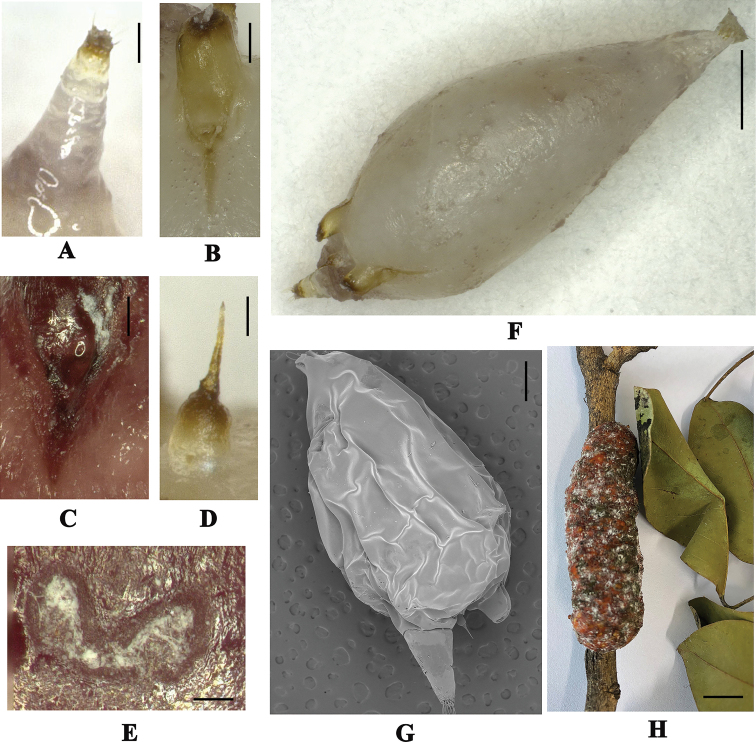
*Kerrianepalensis*. **A** anal tubercle **B** brachia **C** anterior spiracle **D** dorsal spine **E** marginal duct cluster **F, G** body **H** lac tests, ex *Dalbergiacochinchinensis* (**A–F, H** Light micrographs **G** Scanning electron micrographs). Scale bars: 1000 µm (**A**), 200 µm (**B–G**), 1 cm (**H**).

**Adult female**: body generally large globular to elongate in shape, 1.7–3.87 mm long, 1.16–2.42 mm wide (Fig. 1F, G).

***Dorsum*.** Anal tubercle well developed, elongate, 320–1100 µm long, 170–680 µm wide, apparently two-segmented (Figs 1A, 2B) and bearing 6–15 anal ring setae, each 80–90 µm long (Fig. 2A); supra anal plate heavily sclerotized, a little longer than broad, with few small setae on each side (Fig. 2B); brachia oval, elongate (Figs 1B, 2E), heavily sclerotized; brachial plate nearly circular, broader than long; brachial crater circular and small, 80–160 µm long, 70–130 µm wide, 0.03–0.07 mm^2^ in center; brachial tube 210–460 µm long, dimples inconspicuous, uncountable due to thick sclerotization (Fig. 2F); anterior spiracles widely separated (Figs 1C, 2G), 220–400 µm away from brachial plate, canellar bands below anterior spiracles as a chitinous extension 150–300 µm long (Fig. 1B, C); dorsal spine 170–190 µm long, pedicel longer and tubular in shape 80–160 µm long, 70–130 µm wide at widest point (Figs 1D, 2K).

**Figure 2.**
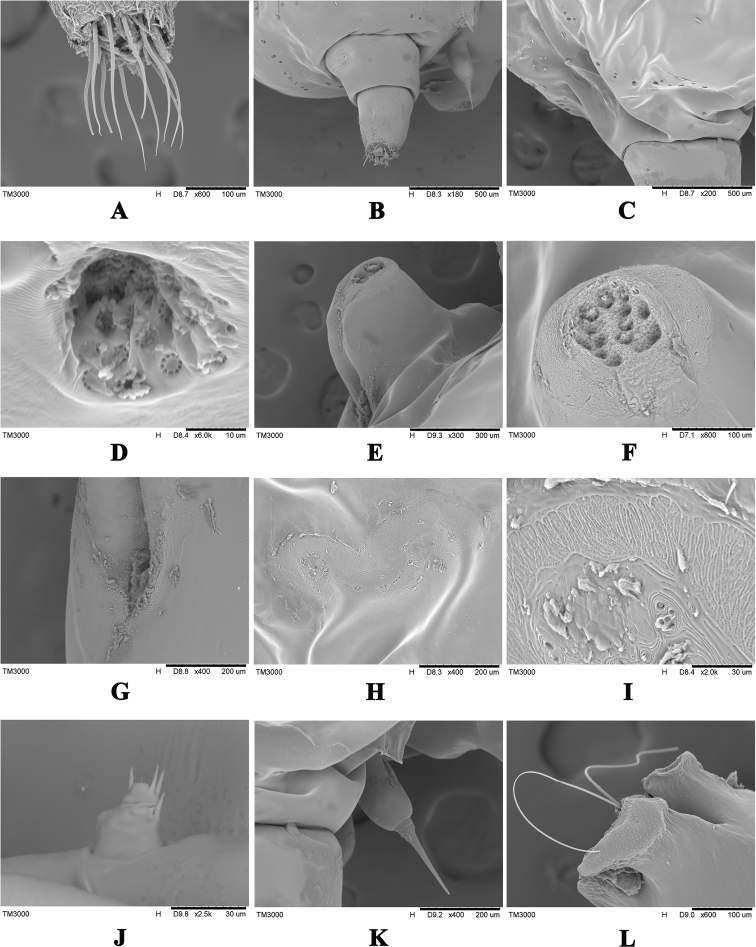
*Kerrianepalensis* scanning electron micrographs **A** anal ring setae **B** anal tubercle and dorsal spine **C** perivulvar pore cluster **D** magnified single perivulvar pore **E** brachia **F** brachial plate with dimples **G** anterior spiracle **H** a marginal duct cluster **I** a magnified marginal duct cluster **J** antenna **K** dorsal spine **L** mouthparts. Scale bars: 10 µm (**D**), 30 µm (**I, J**), 100 µm (**A, F, L**), 200 µm (**G, H, K**), 300 µm (**E**), 500 µm (**B, C**).

***Venter*.** Antennae very small, conical shaped, probably one segmented, with 4 fleshy and 2 short hair-like setae (Fig. 2J); mouthparts with labium length 600–780 µm, width 70–180 µm, post oral lobes each 75–140 µm wide (Fig. 2L); legs vestigial; posterior spiracles much smaller with fine pores on each side; perivulvar pores 14–31 in number on each side of anal tubercle (Fig. 2C, D); marginal duct clusters convoluted (Figs 1E, 2H), 6 in number, each with 30–36 ducts (Fig. 2I); ventral duct clusters with 3 pairs, irregular in shape.

###### Distribution.

India, Myanmar, Nepal (Varshney and Sharma 2020), China (Yunnan).

###### Host plants.

*Dalbergiacochinchinensis* (specimens collected in this study), *Litchichinensis* (Varshney 1976), and *Ficus* sp. (Chen et al. 2011).

### Key to species of the genus *Kerria* from China

**Table:** 

1	Anal tubercle (supra anal plate) elongate, distinctly longer than broad	**2**
–	Anal tubercle (supra anal plate) abbreviated, length subequal to width or broader than long	**6**
2	Canellar pore bands present as a chitinous extension below anterior spiracles	**3**
–	Canellar pore bands absent	**4**
3	Canellar pore bands below anterior spiracles short, 150–300 µm long; dorsal spine 170–190 µm long	***K.nepalensis* Varshney**
–	Canellar pore bands below anterior spiracles very long, 300–500 µm long; dorsal spine 190–240 µm long	***K.chinensis* (Mahdihassan)**
4	Length of brachia subequal or shorter than length of supra anal plate	***K.chamberlini* Varshney**
–	Length of brachia distinctly greater than length of supra anal plate	**5**
5	Supra anal plate smooth; brachial plate with 10–12 distinct dimples; each marginal duct cluster with 25–30 ducts	***K.lacca* (Kerr)**
–	Supra anal plate hispid; brachial plate with 8–15 indistinct dimples; each marginal duct cluster with 30–36 ducts	***K.yunnanensis* (Ou & Hong)**
6	Each marginal duct cluster with 70–75 ducts; distance between anterior spiracle and brachial plate 17–34 μm	***K.mengdingensis* Zhang**
–	Each marginal duct cluster with more than 20 ducts; distance between anterior spiracle and brachial plate greater than 34 μm	**7**
7	Brachial plate diameter equal or greater than length of supra anal plate	**8**
–	Brachial plate diameter distinctly less than length of supra anal plate	**10**
8	Brachial tube 65–90 µm long; anterior spiracles 180–260 µm long	***K.ruralis* (Wang, Yao, Teui & Liang)**
–	Brachial tube 170–340 µm long; anterior spiracles 130–180 µm long	**9**
9	Brachial crater not in center of plate, found near the margin; dimples obscure and small; crater rim open	***K.sindica* (Mahdihassan)**
–	Brachial crater in center of plate; dimples large and distinct; crater rim closed	***K.fici* (Green)**
10	Brachial crater not well defined; number of perivulvar pore clusters 68–70	***K.rangoonensis* (Chamberlin)**
–	Brachial crater well defined; number of perivulvar pore clusters less than 60	**11**
11	Marginal duct clusters duplex, with large nuclear ducts; number of perivulvar pore clusters 58	***K.greeni* (Chamberlin)**
–	Marginal duct clusters simplex, no large nuclear ducts present; number of perivulvar pore clusters less than 50	***K.meridionalis* (Chamberlin)**

## Discussion

*Kerrianepalensis* was identified and described on host *Litchichinensis* from India and Nepal by Varshney (1976). Later it was also recorded from Myanmar (Chen et al. 2011), where it was used for commercial lac production. This species is present in tropical monsoon climates with an average annual precipitation of 800–1000 mm, temperature of 23–29 °C, and at low elevations about 200 m (Chen et al. 2011). *Litchichinensis* (Sonn.) and *Ficus* sp. were the known host plant of *K.nepalensis* (Chen et al. 2011; Varshney and Sharma 2020). We here report *Dalbergiacochinchinensis* as a host of *K.nepalensis*. *Dalbergiacochinchinensis* Pierre ex Laness. is commonly known as Siam Rosewood or Rosewood (Sriudorn and Benchawattananon 2018). It prefers sandy-clay soil, where the mean annual rainfall is 1200–1650 mm and the temperature ranges from 20–32 °C (So et al. 2010; Phunchaisri et al. 2019). It is a perennial tree and distributed in China (Yunnan province), Cambodia, Laos, Thailand, and Vietnam (He 2014; Liu et al. 2016).

The presence of *K.nepalensis* in Yunnan province increases the number of known *Kerria* species in China that could be used for lac production. The natural lac-plant resources are abundant in Yunnan Province (Chen et al. 2010). The Chinese diversity of the genus *Kerria* needs further investigation, and taxonomic studies particularly in Oriental China promise to find new species and new country records of this genus.

## Supplementary Material

XML Treatment for Kerria nepalensis
